# Diagnostic challenges in patients with Castleman disease, a single center experience from Hungary

**DOI:** 10.3389/pore.2024.1611785

**Published:** 2024-08-26

**Authors:** Boglárka Brúgós, Zsófia Simon, Gábor Méhes, Árpád Illés, György Pfliegler

**Affiliations:** ^1^ Division of Hematology, Department of Internal Medicine, University of Debrecen, Debrecen, Hungary; ^2^ Center of Expertise of Rare Diseases, University of Debrecen, Debrecen, Hungary; ^3^ Department of Pathology, University of Debrecen, Debrecen, Hungary

**Keywords:** unicentric Castleman disease, multicentric Castleman disease, interleukin-6, siltuximab, rituximab

## Abstract

Castleman disease is a rare and atypical lymphoproliferative disorder characterized by diverse clinical manifestations. It has both unicentric and multicentric forms, the latter with further subdivisions, i.e., human herpesvirus 8-associated and idiopathic forms. The diagnosis of Castleman disease is often delayed, as it is rare, and because it shares clinical features with different autoimmune, inflammatory, and malignant lymphoproliferative disorders. The first-line treatment in unicentric form is mainly surgical, while in idiopathic Castleman disease, anti-interleukin-6 treatment is the therapy of choice. In virus-associated diseases, antiretroviral therapy and rituximab are recommended. In Hungary, only a few cases of Castleman disease have been published. This report presents our two decades of experience in the challenging diagnosis and management of this rare disorder, most properly underdiagnosed in Hungary. We provide insights into seven unicentric and five idiopathic multicentric Castleman disease cases, the latter ones especially highlighting the diagnostic and therapeutic challenges due to the variable and unique clinical features both of patients and diseases, e.g., bronchiolitis obliterans, stage IV diabetic renal failure, anti-HBc positivity, siltuximab treatment period, respectively.

## Introduction

Castleman disease (CD) is a rare lymphoproliferative disorder with several names, the most widely used “angiofollicular lymph node hyperplasia” [1, 2]. The exact annual incidence remains uncertain. In the United States, idiopathic multicentric Castleman disease (iMCD) incidence and prevalence were reported to be 3.4 and 6.9 per million in 2017–2018 [3]. To our present knowledge, CD has no ethnical or race preferences, which means that, when compared to the populations of the two countries, in Hungary, the annual incidence should be around 50 new cases. However, only 4–6 cases per year are reported, suggesting that it is underdiagnosed.

CD is classified into two main types: unicentric (UCD) and multicentric (MCD) Castleman disease. The latter is further subdivided by the Castleman Disease Collaborative Network (CDCN) based on etiology and includes human herpesvirus 8/Kaposi sarcoma-associated herpesvirus (HHV8/KSHV-associated) and idiopathic MCD (iMCD) [1].

In HHV-8-associated disease, the uncontrolled viral signal, infecting B-cells, and plasmablasts are responsible for cytokine overproduction and systemic symptoms [4]. In these cases, both the human interleukin-6 (IL-6) and viral IL-6 play an important role in B-cell proliferation, mainly because the viral IL-6 binds directly to the IL-6 receptor without its coreceptor (gp80) [5, 6].

Idiopathic MCD has at least four clinical subgroups: POEMS (polyneuropathy, organomegaly, endocrinopathy, monoclonal plasma cell disorder, skin changes) associated MCD, iMCD-TAFRO (thrombocytopenia, ascites, reticulin fibrosis, renal dysfunction, organomegaly), iMCD-IPL (iMCD idiopathic plasmacytic lymphadenopathy), and iMCD-not otherwise specified (iMCD-NOS) [2]. The latest 2022 WHO-HAEM5 classification characterizes CD as a tumor-like lesion with B-cell predominance (Table 1.) [7]. POEMS-associated MCD is caused by the cytokine production from the affected monoclonal plasma cells, which have undergone genomic events [4]. The assumed cytokines in the pathogenesis are vascular endothelial growth factor (VEGF), IL-6, IL-12, transforming-growth factor-1β, and tumor necrosis factor-α.

**TABLE 1:** B-cell lymphoid proliferations and lymphomas [7].

WHO classification 5th edition	WHO classification 4th edition
Tumor-like lesions with B-cell predominance	
Reactive B-cell-rich lymphoid proliferations that can mimic lymphoma	Not previously included
IgG4-related disease	Not previously included
Unicentric Castleman disease	Not previously included
Idiopathic multicentric Castleman disease	Not previously included
KSHV/HHV8-associated multicentric Castleman disease	Multicentric Castleman disease

The pathogenesis of idiopathic MCD is multifactorial [8]. One proposed mechanism is autoimmunity; auto-reactive antibodies stimulate the synthesis and release of cytokines since the histology of CD lymph nodes is 15%–30% similar to lymph nodes found in patients with rheumatoid arthritis (RA) and systemic lupus erythematosus (SLE) [9, 10]. Autoinflammatory syndromes also could share similarities with some iMCD cases. French authors have found an association between the mutation in the Mediterranean fever gene and CD [11]. Moreover, Stone et al. have shown a polymorphism in the IL-6 receptor of iMCD patients, leading to increased levels of IL-6 receptor, thereby IL-6 activity [12]. The fourth proposed mechanism is the neoplastic, which assumes that the development of iMCD is due to acquired oncogenic mutation in lymph nodes [13]. The role of viral infection beyond HHV-8 infection was also proposed as a driving mechanism in iMCD patients (Epstein-Barr virus, HHV-6, hepatitis B virus, Cytomegalovirus, Toxoplasma, etc.) [4].

Clinical manifestations of MCD are systemic inflammation, flu-like symptoms, night sweats, fever, skin rash, edema, and pleural effusions, along with other common symptoms (hepato-splenomegaly) and laboratory abnormalities (high sedimentation rate, increased C-reactive protein, hypoalbuminemia, thrombocytopenia or thrombocytosis). The time to diagnosis of patients with MCD is long not only because of its rarity but the broad spectrum of symptoms mimicking systemic autoimmune diseases, infections, or malignancies. The diagnosis beyond clinical symptoms is based on imaging and histological examination of the lymph nodes, thus the role of the pathologist is decisive.

The 5-year survival of patients with MCD is around 50%–77% [14], while for those with UCD is 100%.

If MCD is suspected, HHV-8 serology is recommended, but it is most commonly developing in immunocompromised patients (also HIV-associated) [1]. Specific biomarkers are not known in iMCD patients, and despite the important role of interleukin-6 (IL-6), it is not necessarily elevated [1].

Fajgenbaum et al. formulated the consensus criteria for iMCD in 2017 [1]. Based on their study *major criteria* of the disease are enlarged lymph nodes and histopathologic lymph node features such as atrophic germinative centers, vascularity, “lollipop” appearance, plasmacytosis, etc. *Minor criteria* are divided into clinical and laboratory criteria. Clinical criteria include “1” constitutional symptoms (fever, night sweats, weight loss, fatigue), “2” large spleen and/or liver, “3” fluid accumulation (edema, anasarca, ascites, pleural fluid), “4” eruptive cherry hemangiomatosis, “5” lymphocytic interstitial pneumonitis. The laboratory criteria include: “1” increased CRP and high sedimentation rate, “2” anemia, “3” thrombocytopenia or thrombocytosis, “4” hypoalbuminemia, “5” renal dysfunction or proteinuria, “6” polyclonal hypergammaglobulinemia [1]. Moreover, infections (HHV-8, HIV, EBV, CMV, toxoplasmosis, tuberculosis), autoimmune and autoinflammatory diseases (systemic lupus erythematosus, rheumatoid arthritis, adult-onset Still disease, autoimmune lymphoproliferative disease) and lymphoproliferative disease (lymphoma, multiple myeloma, plasmacytoma) should be excluded (*exclusion criteria*) [1].

The UCD forms are mainly hyaline vascular type on histology; only a minority of cases are plasmacytic. The lymphoid follicles are abnormal, and the mantle cell lymphocytes surrounding the follicle give the typical onion-skin-like appearance with radially penetrating blood vessels (lollipop appearance). The lymph nodes from MCD patients have three main histological types: hypervascularized, plasmacytic, and mixed type [15].

Surgery is the first-line treatment in UCD. However, there are cases where surgical removal is not feasible because of location or size, and these forms are called unresectable UCD [16]. The latter is further divided into asymptomatic and symptomatic forms. The asymptomatic unresectable UCD must be regularly followed, while in symptomatic forms, debulking, embolization, radiotherapy, or even systemic treatment are recommended [16] using corticosteroids, rituximab, or anti-IL-6 treatment.

Idiopathic MCD and intermediate forms (clinical picture falling between UCD and iMCD) are treated with anti-IL-6 treatment, siltuximab (monoclonal antibody against IL-6) or tocilizumab (monoclonal antibody inhibiting IL-6-receptor). The effectiveness of siltuximab was established in different clinical trials; in an open-label extension analysis of two trials, the patients received 11 mg/kg siltuximab every 3 weeks for up to 6 years [17], and the treatment was well tolerated. The half-life of siltuximab is 20.6 days. In some cases, administration may be extended to 6 weeks. Common side effects were nausea, vomiting, respiratory tract infection, hypertriglyceridemia, arthralgia, and headache; no serious adverse event occurred [18].

Rituximab also can be used in patients with mild symptoms. Siltuximab is recommended to be used continuously, while in non-responders, other immunosuppressive treatments (thalidomide, cyclosporine A, sirolimus, anakinra, bortezomib) are optional. In severe cases combined chemotherapy is appropriate: R-CHOP (rituximab, cyclophosphamide, doxorubicin, vincristine, prednisone, R-VDT-PACE (rituximab, bortezomib, dexamethasone, thalidomide, cisplatin, doxorubicin, cyclophosphamide, etoposide), or etoposide/cyclophosphamide/rituximab [19].

Rituximab and antiretroviral therapy should be used in HHV8/HIV-associated MCD [20] since siltuximab does not bind to viral IL-6 formed in these patients [18].

UCD could be associated with bronchiolitis obliterans and paraneoplastic pemphigus (PNP) which can occur if the treatment is delayed. MCD could increase the risk of thromboembolic complications and could progress into non-Hodgkin lymphomas. In the 2008 WHO classification, a new entity was proposed, the large-B-cell associated HHV-8 positive multicentric Castleman disease which is an aggressive disease caused by the proliferation of HHV-8 infected plasmablasts [21].

*Response criteria* of CD were defined by the CDCN based on laboratory, lymph node, and clinical response. Complete response (CR) means that there is a recovery in all three above-mentioned parameters. Partial response (PR) by definition is improvement in all four symptom categories (anorexia, fever, weight change, fatigue) and improvement in more than 50% of laboratory tests, stable disease is defined as <50% improvement in laboratory tests, and improvement in at least one of each symptom. Progressive disease is more than 25% worsening in laboratory tests and any symptoms [19].

At present, we aim to review the data and clinical characteristics of patients treated for Castleman disease at a Hungarian rare disease and hematology center.

## Material and methods

We performed a retrospective analysis in the Department of Internal Medicine Division of Hematology and Rare Disease Center of University of Debrecen (UD) over 20 years, from 2002 to 2022. Patient records were thoroughly reviewed using the electronic registries (Medsolution and UDMed) of the University of Debrecen. We evaluated demographic information, clinical presentations, histopathologic findings, treatment approaches, and patient outcomes. Histopathologic examinations were primarily performed by the Department of Pathology at UD, and in some cases, a second opinion was sought.

The data analyzed in this study were obtained with the approval of the Regional and Institutional Ethics Committee (RKEB.6437-2023).

## Results

The mean age of all patients was 41.5 ± 18.1 years. We diagnosed and followed seven patients with UCD and five patients with MCD, all of them fulfilled the diagnosis of iMCD. Demographic data are listed in Table 2.

**TABLE 2:** Demographic data of CD (UCD and iMCD) patients.

	UCD (n = 7)	iMCD (n = 5)
Age (years) Gender (M/F)	32.28 ± 13.47 4/3	54.4 ± 16.68 3/2
Localization	Mediastinum (n = 5) Cervical lymph node (n = 2)	Multicentric
Histology	Hyaline vascular n = 6 Plasmacytic (n = 1)	Hyaline vascular n = 4 Plasmacytic n = 1
Surgery	Lymphadenectomy by mediastinoscopy n = 5 Lobectomy = 1	—
Therapy	Chemoimmunotherapy n = 4	Chemoimmunotherapy n = 5

### UCD patients

The mean age of UCD patients was 32.28 ± 13.47 years. The mean time to diagnosis was 5.57 ± 3.4 months. UCD was defined as the involvement of a single lymph node or extranodal site, diagnosed by physical examination, computed tomography (CT), magnetic resonance imaging (MRI), or positron emission tomography (PET/CT).

**FIGURE 1:**
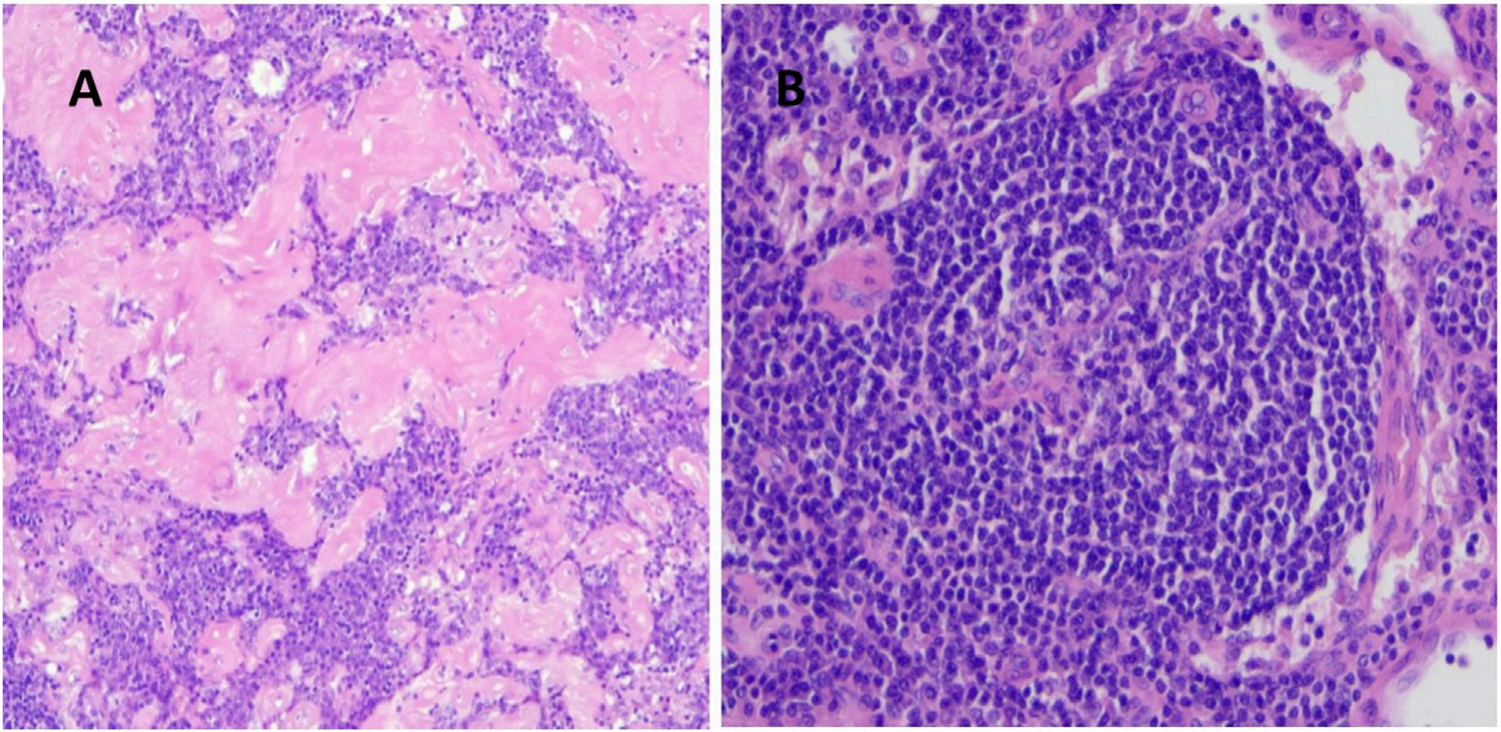
**(A)** Hyalin vascular type histology of a UCD patient (regressed germinal centers, interfollicular vascular proliferation, hyalin deposition, H&E staining magnification ×10), **(B)** umbilical-cord like central vascular pattern (H&E staining magnification ×20).

Most of the patients with UCD had normal inflammatory parameters and complete blood cell counts (CBC) on admission, and all cases were HHV8 negative. Surgical procedures to remove pathological lymph nodes were performed in six patients, with five patients undergoing lymphadenectomy by mediastinoscopy and one patient having right lobectomy as a complication of CD. Histologic examination revealed the hyaline vascular type in most patients (n = 6) (Figure 1) and the plasmacytic type in one patient. In addition to surgery, two patients received thalidomide at doses of 50 and 100 mg for 3 years each. Patients with plasmacytic histology (in 2002) received the CAD protocol (cyclophosphamide 600 mg iv, 10 mg adriamycin, and 40 mg dexamethasone) for four cycles. One patient with mediastinal lymphadenopathy who underwent surgery with hyaline vascular histology (most of the lymphocytes were CD20 positive) received 700 mg rituximab for six cycles, because of unresectable UCD and an increase in inflammatory parameters. At the end of the treatment, PET/CT or chest CT scans showed complete remission in all UCD cases, with a median disease-free remission of 6.83 ± 6.17 years.

### iMCD patients

The mean age of iMCD patients was 54.4 ± 16.68 years. The mean time to diagnosis was 13.6 ± 3.56 months. We diagnosed and followed three male and two female patients with iMCD. The diagnosis of MCD was based on general symptoms, physical examination, CT, MRI, or PET/CT. HHV8 serology was performed in all patients with negative results.

#### Case 1

**FIGURE 2:**
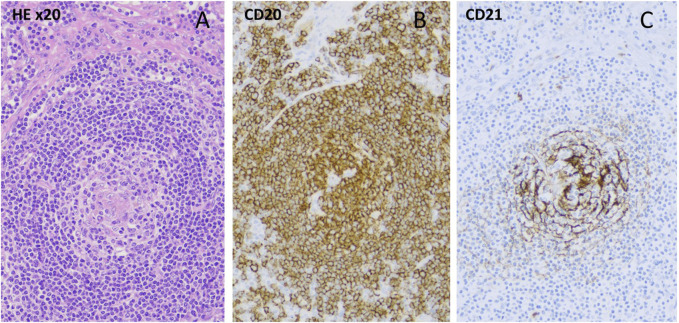
**(A)** Hyalinized centrum germinativum, target plate-like mantle zone/HE ×10, **(B)**-CD20 staining/, **(C)**-loose circular follicular dendritic cell network (CD21 staining).

A 46-year-old woman had a previous history of immune thrombocytopenia that responded well to prednisolone treatment. In 2019, she presented with severe leg edema, livedo reticularis, Raynaud’s phenomenon, vasculitis on the lower leg, hyperpigmentation of the neck, and enlarged axillary lymph nodes. Laboratory tests revealed reduced complement levels and the presence of giant platelets. Tumor markers were negative. Trephine biopsy was performed because prolonged thrombocytopenia showing bone marrow hyperplasia, while information content of axillar and inguinal lymph node core biopsy seemed to be questionable. The renal function was normal, but because of severe leg edema and microscopic hematuria, renal biopsy was performed, showing diffuse mesangial IgM deposition. A skin punch biopsy suggested lupus erythematosus with a positive antinuclear factor (mitotic spindle, titer 1:160). Antibodies to double-stranded deoxyribonucleic acid (DNA), anti-neutrophil cytoplasmic antibody (ANCA), and antiphospholipid antibodies were all negative. Low complement levels were detected (C3 0.38 g/L, C4 0.03 g/L, CH50 < 10). The antiplatelet antibody was positive. Capillary microscopy showed bushy capillaries. In 2022, immunologist excluded systemic autoimmune disease. Therefore, because ultrasonography and CT imaging detected hepatomegaly, enlarged lymph nodes,we decided to get a lymph node biopsy by laparoscopic technique. Based on the clinical symptoms, lymphadenomegaly, laboratory criteria and histology of the lymph node (hyalin vascular CD), we were able to diagnose iMCD (Figure 2), unfortunately with a significant delay. The serum IL-6 concentration was mildly increased, 20 ng/L (N < 7 ng/L). We started the first infusion of siltuximab (11 mg/kg) in January 2023. After the first dose, the blood cell count result showed a marked polycythemia (Hgb 182 g/L, before the infusion it was 165 g/L), which is not a common side effect of siltuximab. Since the possibility of the myeloproliferative disease could not be completely ruled out analysis of Janus kinase-2 (JAK-2) gene- (V617F mutation), JAK2 exon 12-, calreticulin (CALR)-, and myeloproliferative leukemia virus oncogene (MPL) mutations were done with negative results, erythropoietin level was decreased. Phlebotomy was performed twice. After improvement of hemoglobin level, three additional infusions of siltuximab were given without complications. However, after the last infusion, a new enlarged lymph node appeared in the right supraclavicular region, which was removed and histopathology showed CD of the same type. Six weeks after surgery, we restarted siltuximab therapy, which is still ongoing.

#### Case 2

**FIGURE 3:**
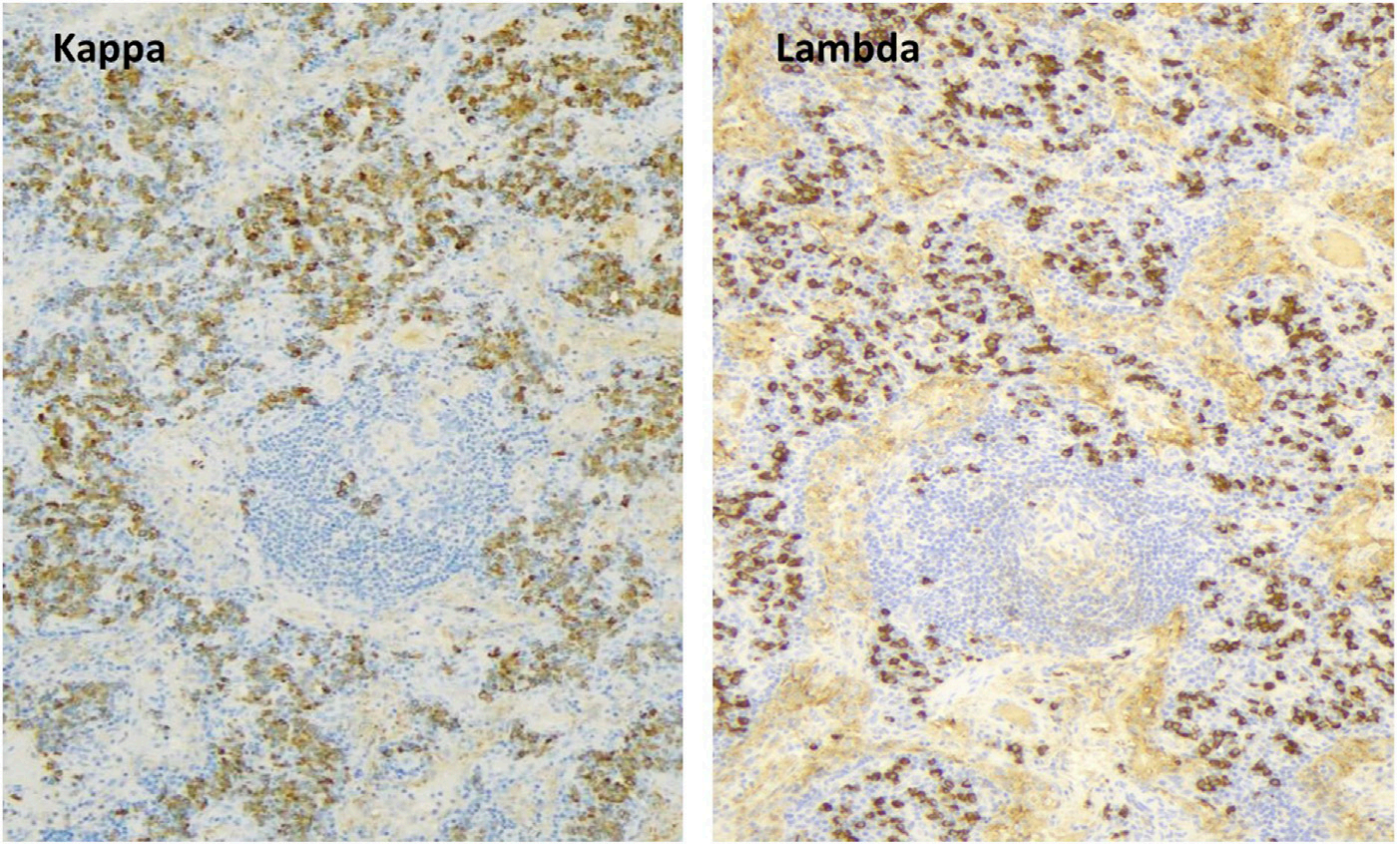
Plasmacytic Castleman disease (degenerative germinal center, plasmocytes, kappa/lambda light chain 2:1).

The 32-year-old woman presented with painful cervical lymphadenopathy, skin lesions diagnosed as paraneoplastic pemphigus based on positive indirect immunofluorescence staining of mouse bladder epithelium, and B symptoms in 2012. AntiBP-180 and antiBP-230 antibodies were also positive, while other tumor markers were negative. Chest and abdominal CT scans showed mediastinal and retroperitoneal lymphadenomegaly, pulmonary nodules, and emphysema. In April 2013, a laparoscopic biopsy confirmed plasmacytic Castleman disease with elevated IgG4 levels (Figure 3). The retroperitoneal mass was removed surgically. The patient also had dyspnea, and spirometry showed severe irreversible central and peripheral airway obstruction (alpha-1-antitrypsin deficiency was excluded). Transbronchial biopsy showed occlusion of small bronchioles, and FEV1 was significantly decreased (17%). Our diagnosis was plasmacytic Castleman disease associated with bronchiolitis obliterans (“popcorn lung”) requiring lung transplantation.

The patient received oral methylprednisolone at a dose of 0.5 mg/kg, budesonide, and tiotropium, as well as six cycles of rituximab (600 mg) for CD. Subsequent PET/CT scans showed metabolic remission, so maintenance rituximab (600 mg) was continued every other month for 1 year. Unfortunately, before the final rituximab infusion, the patient developed an acute respiratory failure and required ventilatory support. No infection was found. Given the known CD, we initiated siltuximab infusion (11 mg/kg), which improved the patient’s clinical condition and allowed the discontinuation of respiratory support. She subsequently received siltuximab infusions every 3 weeks for a total of 16 infusions. Tragically, after the last treatment, she experienced sudden respiratory failure and died at the age of 35.

#### Case 3

A 56-year-old male patient with a history of type 1 diabetes and hypertension presented in 2015 with dizziness, weight loss, and generalized symptoms. Laboratory tests revealed grade 3 anemia and an elevated erythrocyte sedimentation rate (>100 mm/h). Multiple myeloma, systemic vasculitis, and bone marrow involvement were excluded. Abdominal CT revealed retroperitoneal lymphadenopathy and supraclavicular lymphadenomegaly, and a lymph node biopsy was made. The diagnosis was hyaline vascular iMCD since he was HHV8 negative. Methylprednisolone was started in a 1 mg/kg dose, and he received 10 mg of vinblastine every 2 weeks, five times until permission was received for anti-Il-6 treatment, siltuximab. Then siltuximab in a dose of 11 mg/kg was planned; however, due to anti-HBc positivity, antiviral (entecavir) protection (0.5 mg) was required before initiation. Approval was granted to administer 10 cycles of siltuximab (September 2016 to June 2017), during which time the patient remained symptom-free for 3 years and follow-up CT scans showed no lymphadenomegaly.

**FIGURE 4:**
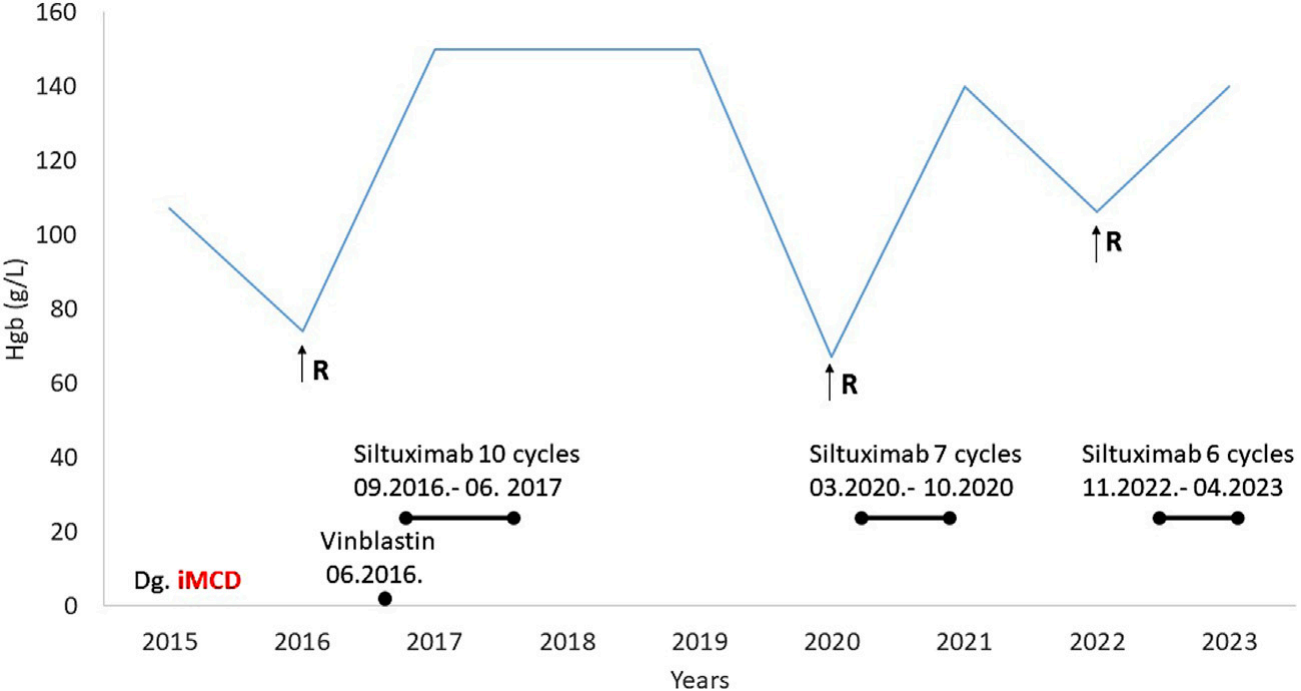
Medical history of Case 3 (R-relapse, Hgb-hemoglobin, MCD-multicentric Castleman disease).

In 2020, anemia-related symptoms recurred, accompanied by lymphadenomegaly and hepatosplenomegaly on abdominal CT. Siltuximab treatment was restarted and continued for seven cycles (Figure 4). The patient experienced a third relapse in November 2022, characterized by lymphadenomegaly and nephrotic proteinuria, along with declining renal function (serum creatinine 229 umol/L, GFR 25 mL/min). However, a renal biopsy confirmed diabetic nephropathy as the cause of renal failure, unrelated to CD. The patient was subsequently treated with six cycles of siltuximab for CD activity. While imaging showed complete remission, there was no improvement in renal function.

#### Case 4

A 76-year-old man with a history of type 2 diabetes, hypertension, and chronic stage IV renal failure (GFR 12 mL/min) presented in January 2022 with anasarca and grade 2 anemia. Chest and abdominal CT revealed generalized lymphadenomegaly (>1 cm), prompting biopsy of an enlarged inguinal lymph node. Histologic analysis confirmed the diagnosis of hyaline vascular CD (iMCD). He was treated with siltuximab for six cycles with no deterioration in renal function. Follow-up imaging showed complete remission.

#### Case 5

A 60-year-old patient with a history of hypertension presented for evaluation of dyspnea and constitutional symptoms in 2015. CT and PET/CT scans revealed generalized lymphadenomegaly and a right lung tumor in the fourth lobe. Lobectomy was performed and histology confirmed HHV8-negative hyaline vascular CD. Treatment with siltuximab was initiated, and of note, the patient has received continuous infusions since 24 September 2015 to date, for a total of 114 siltuximab infusions. Currently, the patient remains in stable disease and no side effects from the infusions have been observed.

## Discussion

Castleman’s disease is a rare lymphoproliferative disorder classified as a tumor-like lesion with B-cell predominance in the new 2022 WHO-HAEM5 classification [7]. The etiology is unknown but is assumed to be related to viral infection (HHV-8), especially in immunocompromised patients, somatic mutations in clonal cells, and autoinflammation [1]. The hypercytokinaemia, the dysregulated cytokine profile, and increased IL-6 level lead to systemic, constitutional symptoms [4].

The unicentric form (UCD) is typically localized to one lymph node region and is often diagnosed incidentally in the absence of general symptoms and normal laboratory tests. Most of the cases could be treated by the surgical removal of the lymph nodes, but in unresectable UCD, especially in those with symptomatic cases systemic treatment is recommended [16]. In our study, five patients had mediastinal and two patients had cervical lymph node enlargement, all malignancies were excluded, and laboratory tests were normal. Surgery was performed in six patients, four of whom received maintenance therapy such as thalidomide, rituximab, prednisolone, or other cytostatic therapy (cyclophosphamide, vinblastine) because of constitutive symptoms despite the lack of morphological signs of lymph node enlargement in other regions. Our median follow-up was 5 years, and the longest disease-free survival was 17 years. Our UCD patients who need systemic treatment may have oligocentric or intermediate CD which is a form between UCD and iMCD [16, 22]. Oligocentric CD is a recently defined type of the disease group which is characterized by regional or neighboring lymph node involvement, which may be asymptomatic, or cause only mild systemic symptoms compared to multicentric form, but sometimes needs further systemic therapy beyond the surgical removal [23]. Based on Turkish observations of a multicentric case series (n = 140), UCD patients received rituximab, radiotherapy, or in a few cases, chemotherapy after surgical removement of the lymph nodes [24].

The diversity of the idiopathic multicentric Castleman disease (iMCD) patients in our study is noteworthy. A unique and fatal combination of plasmacytic CD, bronchiolitis obliterans, severe pulmonary emphysema, and progressive dyspnea occurred in our 32-years-old iMCD patient. Only a few similar cases have been reported in the literature in which the lung disease was complicated by CD-type vascular hyalinosis. The dyspnea improved significantly after removing pathological lymph nodes [25, 26]. This patient needed long in-hospital treatment, similar to the observation of the ACCELERATE study, a longitudinal natural history study of CD. The authors of the mentioned study concluded that iMCD-TAFRO patients needed longer hospitalization than those with iMCD-NOS. Moreover, mechanical ventilation or hemodialysis often became necessary for these patients [27]. The patients under 30 years were more likely to present with severe symptoms [27]. Severe respiratory symptoms were also associated with iMCD in our patient, with the initial symptoms being paraneoplastic pemphigus. Cutaneous manifestations are common in CD as lichenoid eruptions, paraneoplastic pemphigus, maculopapular eruptions, Kaposi sarcoma, cutaneous necrotizing vasculitis, autoimmune bleeding disorders [28, 29]. These cutaneous symptoms occur in 55% of CD patients. The symptoms could be treated with systemic or topical corticosteroids or even with other immunomodulatory treatments.

Our working grouppreviously presented the case of a 30-year-old woman presenting with PNP, the investigation revealed a retroperitoneal mass, and the histology verified the diagnosis of hyalin vascular CD. Considering her young, fertile age, only immunomodulatory treatment with corticosteroid, thalidomide, and cyclosporine-A was started, which led to remission of both the tumor and PNP [30].

Livedo reticularis, Raynaud phenomenon, and lower limb vasculitis in case 2 raised the possibility of antiphospholipid syndrome or systemic lupus erythematosus since the skin biopsy suggested it, but the clinical symptoms did not fulfill the diagnostic criteria of SLE. Nishimura et al. suggested that some patients with iMCD could have undiagnosed autoimmune diseases [31]. They examined retrospectively 63 patients with iMCD, 19 patients had at least one positive autoantibody, and five patients had positive antiphospholipid antibodies. In such patients, immunosuppressive treatment is also recommended, in our iMCD patient we also observed some symptoms suggesting autoimmune disease.

The association of systemic autoimmune disease and CD is rare, but it was shown previously that 30% of iMCD patients in case reports have autoantibodies [4, 31]. In one case report, UCD was associated with mixed connective tissue disease, and in another one TAFRO syndrome (histology was mixed type) was developed in a patient with Sjögren syndrome [32, 33]. Our previously mentioned patient (case 2) had Raynaud-phenomenon, livedo reticularis, low complement level, and antibody against antinuclear factor on skin biopsy, although negative lupus-specific antibodies are also unique symptoms in patients with CD. Association of systemic lupus erythematosus with CD was reported in only three pediatric cases [34].

In most of our cases, no side effects of siltuximab were observed, except in case 2, where the first siltuximab infusion resulted in significant polycythemia (Hgb 181 g/L, Htc 0.55). Therefore, we delayed the next infusion until a mild reduction in hemoglobin level (Hgb 167 g/L)and exclusion of any genetic mutation behind her polycythemia. In her case, secondary polycythemia seemed to be more likely while she has been a 1 pack/day smoker for more than 20 years, which was deteriorated by the use of siltuximab. In a 2018 systemic review that included 171 cases of MCD patients, polycythemia was reported as a side effect of siltuximab treatment in only one case [17, 35].

In case 3, the patient’s disease course included remissions and relapses [36], but siltuximab was effective in each relapse and at least stabilized the disease course. Siltuximab treatment was effective without serious side effects even in cases with stage IV renal failure, as observed in cases 3 and 4. Renal involvement is uncommon in CD. Karoui et al. published a case series with histology-approved renal involvement with CD. 19 CD patients were included, mostly iMCD patients and four cases were HIV-positive. The HIV-negative iMCD patients have mostly small-vessel lesions with endotheliosis and in some cases AA amyloidosis [37]. 50% of patients with TAFRO syndrome have renal dysfunction, and most of the kidney histology shows thrombotic microangiopathy-like pathology and mesangioproliferative-like glomerulonephritis [38]. In case 3 the patient’s renal dysfunction was thought to be caused by a relapse in his Castleman disease, but a renal biopsy revealed diabetic nephropathy behind his nephrotic proteinuria and stage IV renal failure. In case 4 the stage IV renal failure was diagnosed 2 years before the occurrence of CD and was due to his 10-year history of type 2 diabetes mellitus. A remarkable observation in case 5 iMCD patients is the continuous administration of siltuximab for almost 8 years, significantly longer than reported in an extension analysis study with an average duration of 5.5 years [17]. The maximum duration of the use of siltuximab is not established. In a previous case report, Lang et al. have been using siltuximab for 15 years as a maintenance therapy [39], and they found it to be safe in such long-term treatment.

The median survival of our iMCD patients was 4 years which is comparable to the average survival of the patients published previously, 25%–45% of them die within the first 5 years, and patients often relapse within 1–2 years [19]. In a phase II clinical trial siltuximab had a 34% overall response rate [40].

The diagnosis and treatment of iMCD are challenging [41] since it shares symptoms similar to different chronic inflammatory diseases. In milder cases, glucocorticoids might be enough, but in severe cases targeted therapy as anti-IL-6 treatment, or the use of combined chemotherapy could be optional.

Considering the high mortality rates of the iMCD patients new, targeted therapies are needed to improve the survival rate.

We recognize our study’s limitations, this was a retrospective single-center review of a rare disease with a limited sample size. The small sample size and the incomplete, reliable data were not sufficient to make a serious statistical analysis. Multicenter data with long-term follow-up can improve our results.

## Conclusion

Castleman disease is a rare and atypical lymphoproliferative disorder that often presents with symptoms similar to other malignant lymphoproliferative and autoimmune or inflammatory disorders. As a result, an accurate diagnosis might be delayed, sometimes for several months or years. A key feature of the disease is lymphadenomegaly, making lymph node biopsy and histologic examination essential for accurate diagnosis. The unicentric form typically follows a benign course and usually requires no systemic treatment beyond lymph node sampling. In contrast, the multicentric variants of Castleman disease both the HHV8-positive cases and iMCD are associated with generalized symptoms and laboratory abnormalities which require systemic treatment, primarily anti-IL-6 therapy. Given the rarity of the disease, the diagnosis is challenging and the role of histological diagnosis cannot be questioned.

## Data Availability

The raw data supporting the conclusions of this article will be made available by the authors, without undue reservation.
